# Have “new” methods in medical education reached German-speaking Central Europe: a survey

**DOI:** 10.1186/1472-6920-14-172

**Published:** 2014-08-16

**Authors:** Martin Fandler, Marion Habersack, Hans P Dimai

**Affiliations:** 1 Department of Emergency and Critical Care Medicine, Nuremberg Hospital, Prof.-Ernst-Nathan-Str., 1, 90419 Nuremberg, Germany; 2 Office of the Vice Rector for Teaching & Studies, Medical University of Graz, Auenbruggerplatz 2, 8036 Graz, Austria; 3 Department of Internal Medicine, Division of Endocrinology & Metabolism, Medical University of Graz, Auenbruggerplatz 2, 8036 Graz, Austria

**Keywords:** Medical education, Emergency medicine, Simulation-based-training, Simulation and training center

## Abstract

**Background:**

Simulation-based-training (SBT) in the education of health professionals is discussed as an effective alternative for knowledge and skills enhancement as well as for the establishment of a secure learning environment, for learners and patients. In the Anglo-American region, SBT and simulation and training centers (STC) are numbered as standard for medical training. In German-speaking Central Europe, priority is still given to the establishment of SBT and STC. The purpose of this study was (i) to survey the status quo relating to the existence and facilities of simulation and training centers at medical universities in German-speaking Central Europe and (ii) the evaluation of training methods, especially in the area of emergency medicine skills.

**Methods:**

All public and private medical universities or medical faculties in Germany (36), Austria (4) and German-speaking Switzerland (3) were interviewed. In the survey, information regarding the existence and facilities of STCs and information with regards to the use of SBT in the area of emergency medicine was requested. The questions were partly posed in a closed-ended-, in an open-ended- and in a multiple choice format (with the possibility of selecting more than one answer).

**Results:**

Of a total of 43 contacted medical universities/medical faculties, 40 ultimately participated in the survey. As decisive for the establishment of a STC the potential to improve the clinical-practical training and the demand by students were listed. Obligatory training in a STC during the first and sixth academic year was confirmed only by 12 institutions, before the first invasive procedure on patients by 17 institutions. 13 institutions confirmed the use of the STC for the further training of physicians and care-staff. Training for the acute care and emergency medicine skills in the field of pediatrics, for the most part, occurs decentralized.

**Conclusions:**

New methods in medical training have reached German-speaking Central Europe, but the simulation and training centers vary in size, equipment or regarding their integration into the obligatory curriculum as much as the number and variety of the offering to be trained voluntarily or on an obligatory basis.

## Background

Simulation-based-training (SBT) in the education and advanced training of health professionals is discussed internationally as an effective alternative for knowledge enhancement, improvement of “behavioral learning outcomes” as well as for technical and team skills [1,2]. Based on theories and concepts of adult education or organizational psychology SBT is merchandized as an alternative – superior to the traditional learning and teaching scenarios – for the establishment of a secure learning environment, for learners and patients [3-5]. In this context many medical specialty fields use SBT (partially in simulation and training centers specifically built for this) in order to guarantee or optimize the quality of the training and, in further consequence, the quality of patient care [6,7]. Minimization of the “July effect” [8], the limitation of the strategy, “Learning by Doing” [9], – regarded critically by the public as well as by patients –or simply the reaction to resource scarcity (minimizing study time or training time, financial and/or staff resources) are listed, among other things, as additional reasons for the establishment of SBT or STCs [10]. The specific requirements for institutions and learners in the context of simulation-based medical education are multifaceted and complex. In that manner, referring to the conception and establishment of SBT or STCs simulation and training centers, not only the provision of learning facilities in general and of clinical equipment, models and manikins in particular, but also the integration of SBT into the curricula or the adoption of innovative teaching and learning methods are to be ensured [11-13]. The consistent application of reliable and valid instruments for the evaluation of simulation learning outcomes and the hesitant application of summative as well as formative assessments is an additional challenge discussed in the literature [5,14,15]. Within the framework of SBT, cognitive skills as well as non-cognitive skills can be imparted. Correspondingly, different assessment instruments ensue for different teaching and learning contents (e.g., performance-based clinical skills, critical thinking/problem-solving skills and/or abilities, behavior- and team interaction). Despite these requirements and challenges, especially in the Anglo-American region, SBT and STCs are numbered as standard for medical training and the optimization of framework requirements, new methodologies and competencies to be taught are demanded. In contrast, in German-speaking Central Europe, as shown in the study by Segarra et al. (2008), priority is still given to the establishment of SBT and STCs [16].

The objective of this study was

a) to survey the status quo relating to the existence and facilities of simulation and training centers (STC) at medical universities/faculties in Germany, Austria and Switzerland; and, presupposed the existence of a STC was affirmed,

b) the evaluation of training methods, especially in the area of emergency medicine skills (general and pediatric).

## Methods

In the period from February 2011 to July 2011, all public and private medical universities or medical faculties (respectively, the university board of these institutions) in German-speaking Central Europe were asked to participate in a survey about the topic of simulation and training centers (STC). The dynamically fillable survey was sent via e-mail to the university board. The participating universities/faculties were assured of the anonymization of all data.

The focus on medical universities/medical faculties in German-speaking Central Europe – at the time of data collection, this included 36 universities/faculties in Germany, four in Austria and three in German-speaking Switzerland – was based on two considerations:

a) avoidance of language barriers and

b) similarity of courses of study in relation to duration and content. In this context, medical college in Germany and in Austria is designed as a 6-year undergraduate program (after high school). After the successful completion of this course of studies/examinations, the degree of Medical Doctor (MD) is awarded in Austria, and students become licensed physicians in Germany. In Switzerland, medical college is also designed with a total length of six years, but with the Bologna-compliant division into a 3-year bachelor program (after high school) and a 3-year master program in clinical medicine. In Switzerland, the course of study is concluded with the Master of Medicine.

The survey focused on two subject areas:

(i) collection of general (administrative) data such as existence, size, financial resources, reasons for the establishment, infrastructure, and integration into the teaching (27 questions) as well as an additional question regarding the specialty fields represented in the simulation and training centers (one question). The wording of the questions in this subject area was in the style of a survey conducted in 2008 by Segarra, et al. (2008) [16],

(ii) collection of general emergency medical skills taught (22 questions) and skills taught particularly regarding pediatric emergencies (nine questions). Wording of questions in this subject area was developed especially for this precise survey and tested regarding their face-validity by an expert team. The expert team was composed of instructors of the institution, experts in the area emergency medicine/pediatrics, the scientific director of the local STC, and upper-semester students.

The questions were posed either in a closed-ended- or open-ended or multiple-choice format (with the possibility of selecting more than one answer).

### Ethics

The work described in this paper was primarily an “administrative data” evaluation and therefore was exempt from requiring permission from the *Ethikkommission der Medizinischen Universität Graz*[17].

## Results

Of a total of 43 contacted medical universities or medical faculties, 40 ultimately participated in the survey. This corresponds to a return rate of 93%.

### General data

#### ***Establishment of STCs/year***

**Figure 1:**
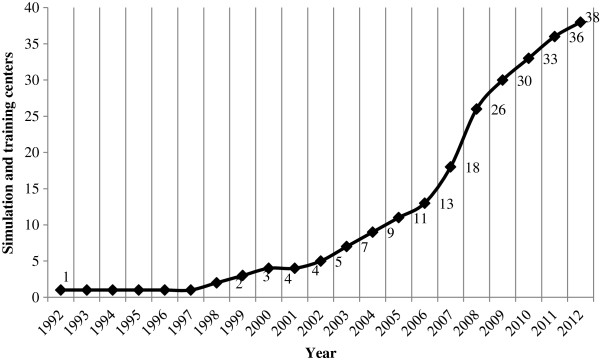
**Establishment of STCs**/**year.**

36 institutions (of a total of 40 medical universities/medical faculties participating in the survey) indicated they had a simulation and training center at their disposal. Two institutions reported the planned establishment of a STC; one institution indicated the existence of a decentralized STC. Only one institution did not confirm the existence or planning of a STC.35 institutions answered the questions regarding an expansion of STCs. Twenty-three of these 35 institutions confirmed the planned expansion of the STC in the next five years (e.g., greater number of phantoms, greater number of stations) and 20 medical universities/faculties indicated they want to increasingly incorporate STC s into the obligatory classes (Figure 1).

#### ***Reasons for/against the establishment and the expansion of STCs***

Reasons that were listed as essential for the establishment of STCs were indicated by 36 medical universities/faculties (multiple responses were possible). Of these 36 institutions, 29 institutions mentioned the potential to improve the clinical-practical training, and respectively 17 centers the potential to minimize deficits in the clinical-practical training as decisive for the establishment of a STC. The demand by students was listed by 22 universities/faculties as decisive for the establishment of a STC. Eleven institutions indicated, with the establishment of a STC they were reacting to trends in medicine didactics.

32 of 40 institutions gave information regarding the reasons for the further expansion of a simulation and training center (multiple responses were possible). As relevant for the expansion of an existing STC, 18 of 32 institutions answering this question listed the need by students. The explicit demand by students for an expansion of the STC was listed by twelve institutions. 25 of 32 institutions mentioned the potential for improving the clinical-practical training as essential reason for the expansion of an existing STC.

The answers to this set of questions are depicted in Table 1 in condensed form.

**Table 1:** Reasons for the establishment and the expansion of STCs

**Reasons for**	**Establishment (n = 36)**		**Expansion (n = 32)**	
Potential to improve clinical training	29	80.56%	26	81.25%
Student demand	22	61.11%	12	37.50%
Teacher demand	9	25.00%	10	31.25%
Department leadership demand	11	30.56%	6	18.75%
Others	4	11.11%	4	12.50%
Planned external cooperation	3	8.33%		
Combined individual projects	9	25.00%		
Existing deficits in clinical training	17	47.22%		
Current facilities not adequate			26	81.25%
Necessity for student training			18	56.25%
Necessity for mandatory training			6	18.75%
Additional budget (from university)			10	31.25%
Additional budget (e.g. sponsoring)			5	15.63%

As reasons against the establishment of a STC (not depicted in the table) lacking financial resources (eleven of 19 responding institutions) and non-existing facilities (14 of 19 responding institutions) were listed. Lacking financial resources were additionally listed as main reason against the expansion of an existing STC (twelve of 16 responding institutions).

#### ***Qualification of STC administrators***

The question which qualification the administrators of the simulation and training centers possess was answered by 37 institutions. Twelve medical universities/medical faculties confirm the administration of the STC by physicians with further training of a Master of Medical Education (MME) or equivalent education; 17 institutions are led by physicians without special academic training in medical didactics. 31 centers (36 responses) use physicians and students as teachers. Only five centers employed, for example, psychologists or certified care givers as teaching staff.

#### ***Financing of STCs***

Regarding the financing of the initial equipment/the construction of a STC and the financing of the running expenses of a STC (multiple responses were possible), a majority of the institutions indicated financing via the college budget (initial equipment: 30/running expenses: 29). Tuition fees were listed by nine institutions as financing source for the initial equipment and by eight institutions for covering running expenses. Only seven institutions use/used private means for financing the construction and financing the running expenses of a STC (Table 2).

**Table 2:** Financing of the initial equipment and of the running expenses of STCs

		**Initial equipment (n = 36)**		**Running expenses (n = 34)**
College budget	30	83.33%	29	85.29%
Private sources	7	19.44%	7	20.59%
Others	11	30.56%	10	29.41%
Tuition fees (included in “others”)	9	25.00%	8	23.53%
Cooperation/sponsorship	5	13.89%	2	5.88%

#### ***Obligatory training in STCs***

The question, whether students are obligated to attend simulation and training centers during the various academic years, was answered by 31 centers. 22 institutions confirmed the obligatory attendance during the third academic year and 21 institutions each during the fourth and fifth academic year. Only at twelve institutions training in STCs is obligatory for students during the first academic year and sixth academic year. Obligatory training in a STC before the first invasive procedure on patients was confirmed by 17 institutions.

Of 36 responding institutions, only 13 institutions confirmed the use of the STC for the further training of physicians and care-staff. The question, whether didactic research is being conducted at the STCs, was answered positively by 20 institutions (37 responses). Nine centers confirmed that future research activities were being planned, and eight centers indicated they neither conducted nor planned didactic research (Table 3).

**Table 3:** Obligatory training in STCs

**Year**	**Obligatory training (n = 31)**	
1	12	38.71%
2	10	32.26%
3	22	70.97%
4	21	67.74%
5	21	67.74%
6	12	38.71%

#### ***Distribution of specialties taught in STCs***

As seen from Table 4, a wide variety of clinical subjects/specialty fields are taught in the STCs. At 32 centers (38 responses, multiple answers were possible) abilities/skills in internal medicine, at 31 centers abilities/skills in surgery, and at 29 centers abilities/skills in emergency medicine are taught. The clinical subjects/specialty fields: gynecology, orthopedics, trauma and orthopedic surgery and urology are offered at 25 simulation and training centers. Medical communication is taught in 24 centers. Only two centers offer topics around medical ethics. Individual centers indicated additional subject areas such as medical English, forensic medicine, psychosomatic medicine, geriatric medicine or micro surgery as classes (not depicted in the table).

**Table 4:** Distribution of specialties

		**Specialties (n = 38)**
Internal medicine	32	84.21%
Surgery	31	81.58%
Emergency medicine	29	76.32%
Anesthesiology/critical care	26	68.42%
Obstetrics/gynaecology	25	65.79%
Trauma-/orthopaedic surgery	25	65.79%
Urology	25	65.79%
General-/family medicine	24	63.16%
Medical communication	24	63.16%
Neurology	22	57.89%
Otolaryngology	19	50.00%
Pediatrics	19	50.00%
Radiology	14	36.84%
Ophthalmology	13	34.21%
Pneumology	12	31.58%
Psychiatry/psychology	8	21.05%
Medical ethics	2	5.26%

### Emergency medical abilities

The second range of topics in the questionnaire focused on emergency medical abilities and skills that are taught at the STCs. 22 skills in the general and 9 skills in the area of pediatric acute care and emergency medicine were enquired about. Medical universities/medical faculties participating in this collection of data could choose four answers (multiple responses were possible): a) skill is taught in the STC in the frame work of obligatory subjects, b) skill can voluntarily be trained by students in the STC, c) the establishment of appropriate training possibilities is being planned, and d) the skill is taught outside the STC.

#### ***General emergency medicine skills***

In the area of general emergency medicine skills, 23 institutions indicated they offer, for example, training for advanced life support (according to the European Resuscitation Council, ERC) and the use of automatic defibrillators in the STC in the frame work of the obligatory subjects. Voluntary training for a simple cutaneous suture or the placing of a central venous access was listed by 17 institutions. The planning of appropriate training possibilities with regards to the application of dressings (including compression bandage) and the interpretation of radiological emergency findings (fracture, intra-cranial bleeding, etc.) was affirmed by six institutions. According to the information given by the centers, training for the internal (16 institutions), the neurological (17 institutions) and the surgical emergency check (15 institutions) is performed most frequently in a decentralized manner, that is, at the respective departments themselves. 21 universities/faculties indicate that the placement of a peripheral venous access, stabilization of the cervical spine and the interpretation of an emergency ECG’s (including STEMI/NSTEMI) are taught in the STCs within the framework of the obligatory subjects. Voluntary training is offered in the STCs for placing an arterial access (15 centers), bag-valve ventilation (twelve centers) or advanced trauma care (ATLS or ETC or comparable alternatives) (eleven centers) (Table 5).

**Table 5:** General emergency medicine skills

	**n**		**Mandatory**		**Voluntary**		**Planned**		**Decentralized**
Focused examination (internal medicine)	32	13	40.6%	10	31.3%	4	12.5%	16	50.0%
Focused examination (neurology)	29	9	31.0%	6	20.7%	4	13.8%	17	58.6%
Focused examination (trauma)	31	13	41.9%	8	25.8%	3	9.7%	15	48.4%
IV access, peripheral	37	21	56.8%	15	40.5%	4	10.8%	7	18.9%
IV access, central	29	8	27.6%	17	58.6%	4	13.8%	7	24.1%
Arterial line	31	7	22.6%	15	48.4%	5	16.1%	8	25.8%
Bag-valve ventilation	35	24	68.6%	12	34.3%	1	2.9%	15	42.9%
Orotracheal intubation	35	25	71.4%	9	25.7%	2	5.7%	14	40.0%
Alternative airway devices (LT, LMA etc.)	34	22	64.7%	7	20.6%	3	8.8%	14	41.2%
Wound dressing	33	16	48.5%	12	36.4%	6	18.2%	10	30.3%
Simple cutaneous sutures	37	21	56.8%	17	45.9%	5	13.5%	14	37.8%
Splinting of extremities	31	16	51.6%	7	22.6%	2	6.5%	14	45.2%
C-spine stabilization	33	21	63.6%	8	24.2%	1	3.0%	11	33.3%
Extrication of injured patient	31	19	61.3%	6	19.4%	2	6.5%	10	32.3%
AED usage	34	23	67.6%	6	17.6%	2	5.9%	12	35.3%
Defibrillator- (manual, semi-manual) usage	33	25	75.8%	5	15.2%	2	6.1%	11	33.3%
Emergency ECG interpretation	34	21	61.8%	10	29.4%	2	5.9%	11	32.4%
Emergency radiology interpretation	30	6	20.0%	8	26.7%	6	20.0%	18	60.0%
Trauma emergency sonography (FAST etc.)	24	5	20.8%	6	25.0%	3	12.5%	13	54.2%
Advanced trauma care (ATLS, ETC etc.)	27	8	29.6%	11	40.7%	4	14.8%	12	44.4%
Basic life support (ERC/AHA)	35	26	74.3%	6	17.1%	2	5.7%	13	37.1%
Advanced life support (ERC/AHA)	35	23	65.7%	6	17.1%	3	8.6%	13	37.1%

#### ***Acute care and emergency medicine skills: pediatrics***

Training for the acute care and emergency medicine skills in the field of pediatrics, for the most part, occurs decentralized, that is, at the respective clinics. Students can train pediatric bag-valve ventilation, intubation of children with a laryngoscope and alternative airway devices (larynx tube, etc.) in twelve STCs on a voluntary basis. In the frame work of obligatory classes, for example, pediatric basic life support (ERC) is taught in STCs in twelve institutions, and the placement of intraosseous access or the examination of a pediatric emergency patient in seven institutions in STCs (Table 6).

**Table 6:** Acute care and emergency medicine skills: pediatrics

	**n**		**Mandatory**		**Voluntary**		**Planned**		**Decentralized**
Pediatric basic life support	30	12	40.0%	9	30.0%	3	10.0%	12	40.0%
Pediatric advanced life support	26	8	30.8%	9	34.6%	3	11.5%	11	42.3%
Focused examination (pediatrics)	24	7	29.2%	4	16.7%	5	20.8%	17	70.8%
IV access, peripheral	24	3	12.5%	8	33.3%	2	8.3%	14	58.3%
Alternative application of medication (rectal, buccal, nasal, intramuscular)	22	3	13.6%	6	27.3%	1	4.5%	15	68.2%
Intraosseous access	23	7	30.4%	8	34.8%	3	13.0%	10	43.5%
Bag-valve ventilation	28	9	32.1%	12	42.9%	2	7.1%	15	53.6%
Orotracheal intubation	25	7	28.0%	12	48.0%	1	4.0%	12	48.0%
Alternative airway devices (LT, LMA etc.)	28	8	28.6%	12	42.9%	3	10.7%	13	46.4%

## Discussion

The objective of the current study was to survey the status quo of simulation and training centers at medical universities/medical faculties in Germany, Austria and German-speaking Switzerland, and to gain an overview of offered emergency medicine training methods/activities. Of 43 contacted medical universities/medical faculties, 40 institutions ultimately participated in the data collection. The results of the investigation document firstly the rising number of STCs as well as the definite commitment to the expansion of STCs in German-speaking Central Europe. Whereas in the year 2007 simulation and training centers existed at 13 medical universities/medical faculties, already 38 medical universities confirmed the establishment of a STC in 2012. This “trend” was already diagnosed by the results of Segarra et al. (2008), and – at least for Germany – attributed to the reform of the medical licensure law in the year 2002 and to the levy of tuition fees in the year 2006 [16]. The establishment of STCs or simulation-based education before this time can, in an international comparison, be interpreted as unassertive [18]. As essential reasons for the construction or the expansion of a simulation and training center the polled medical universities / medical faculties listed the potential to improve clinical-practical training, deficits in the training, didactic considerations, and the explicit demand by students. This corresponds to the areas mentioned in numerous publications in which simulation-based training is mentioned as a possibility for change, improvement or development. In this context, Bradley et al. (2003) list the areas: fundamental reforms in medical education, the “adoption” of the concept of clinical governance, deficits in medical training and the potential to answer to altered requirements with adequate course offerings [11,19,20]. The safety and optimization of patient care are listed as further areas that argue for simulation-based medical education [2,20,21].

The polled centers mentioned the lack of financial resources as essential reason speaking against the establishment or expansion of a STC. The literature, as well, refers to the not insubstantial costs of a simulation and training center and considers these, among other things, also responsible for the hesitant implementation of the same [5,18,22,23]. Those points of time in the framework of medical training at which obligatory training in a STC is stipulated are doubtlessly associated with patient safety. The results from the data collection show that this “obligation”, on one hand, exists before clinical training and, on the other hand, in the fourth or fifth academic year. This corresponds to the implementation of (high-fidelity) simulator exercises, common internationally, in the pre-clinical as well as in the clinical part of the curriculum [24]. McGaghie et al. – in their qualitative synthesis published in the year 2010 – furthermore refer to the importance of and also the difficulties with the integration of simulation-based medical education in curricula [15]. Currently – as the results of the present data collection show – the STCs are predominantly used for students. This does not correspond to the results of numerous studies that point out the definitely positive effect of simulation-based education on the clinical skills of post-graduates [25,26].

As Table 4 in the results section shows, a variety of subjects is taught in the simulation and training centers in Germany, Austria and German-speaking Switzerland. Internal medicine, surgery as well as emergency medicine, as cross-sectional area, are offered most frequently.

The results from the second range of topics in the questionnaire – where general emergency medicine skills and emergency medicine skills in the area of pediatrics were the focus– show a great variability in the “offering”. Classic emergency medicine skills are frequently offered in the framework of obligatory subjects in the STCs; however, the number of skills offered to be trained voluntarily is low. This seems all the more surprising as definite indications already exist for the fact that simulation-based medical education could – among other things, in the area of emergency medicine –minimize potential complications, increase trainee safety, or simply could, as Singer et al. (2013), among others, document, lead to better results for residents (with simulation-based training) in comparison to residents without simulation-based training [27].

Emergency medicine skills in the area of pediatrics are rarely offered in the polled STCs in the context of obligatory classes, but would– though not prevalent –in no case be a negligible tool [28]. Though, as Kessler et al. (2013) indicate, there are few studies on whether SBT has an improved clinical impact on pediatric patients, among other things, the exceedingly positive effects of SBT in the area of echocardiography in congenital heart diseases with children is pointed out [29]. In any case, one has to assume that, even in the context of pediatric emergency medicine, parents and/or relatives as well as residents would welcome comprehensive SBT [26].

In summary, it can be noted that the STCs in German-speaking Central Europe vary strongly in their facilities, their resources, in their utilization, in their offerings, and in the qualification of the teachers/instructors. In the words of Beckers et al. (2009), “The innovative options of simulation technology or state-of-the-art assessment methods are not consistently utilized” [13]. Admittedly, recommendations exist developed by and for various medical/care-giving specialty fields.

For Germany, the efforts of the German Medical Faculty Association and the German Medical Association (GMA) to create a Germany-wide competency-based catalogue of learning objectives for undergraduate medical education, will not only define the levels needed to be taught to students but also further the importance of SBT and associated STCs [30]. This might be a way to promote the same basic level of emergency medical education and SBT at all German universities. Also, this catalogue could be a model for Austria, where no nation-wide competency-based catalogue currently exists.

However, currently there are no minimal standards for SBT und STCs applicable to all of Europe. The agreement on minimal standards applying to all of Europe (the results of the EU project *Simbase*, among others, would have to be valued as a possible starting point for this), in turn have to be interpreted as potential starting point for an Europe-wide accreditation of STCs [31]. Minimal standards – as they are planned, e.g., internationally by the Society for Simulation and Health Care for the accreditation of STCs – would be, not least, essential for a barrier-free mobility for students and work in Europe [32]. In this context, the Europe-wide ascertainment of the status quo of SBT and STCs would have to be assessed as first step towards formulating minimal standards.

### Limitations

Though the present survey represents the status quo regarding SBT and STC at medical faculties in German-speaking Central Europe almost completely (response rate 93%), the inclusion of all European countries in a survey concerning this matter is a next necessary step.

## Conclusions

In summary, one gets the impression that new methods in medical training have reached German-speaking Central Europe, but that the simulation and training centers vary greatly in size, equipment or regarding their integration into the obligatory curriculum as much as the number and variety of the offering to be trained voluntarily or on an obligatory basis. Still, questions about financial feasibility and about the “necessity” (teachers versus students) remain in the foreground and further complicate the – already hesitant, in international comparison – development or the realization of a systematic assessment of the outcomes. The limitation of the training offering to students (i.e., graduated physicians or other health professionals are rarely addressed) and only minimal collaboration with other institutions in health care, on no account, argue for a possible termination of “financial crises”.

However, the number of existing simulation and training centers in German-speaking Central Europe can be seen as indication that the importance of SBT for the quality of medical training and patient care was recognized.

## Abbreviations

AED: Automated external defibrillator; AHA: American heart association; ALS: Advanced life support; ATLS: Advanced trauma life support; ECG: Electrocardiogram; ENT: Ear, nose, throat (otolaryngology); ERC: European Resuscitation Council; ETC: European trauma course; FAST: Focused assessment with sonography in trauma; IM: Intramuscular; IV: Intravenous; LMA: Laryngeal mask airway; LT: Laryngeal tube; MME: Master of medical education; NSTEMI: Non-ST-elevation myocardial infarction; OB/GYN: Obstetrics/gynaecology; SBT: Simulation-based-training; STC: Simulation and training center; STEMI: ST-elevation myocardial infarction.

## Competing interests

The authors declare that they have no competing interests.

## Authors’ contributions

MF participated in the design of the study, performed the data collection and analysis and revised the manuscript critically. MH contributed in the interpretation of data and drafted the manuscript. HPD participated in the design of the study and helped to draft the manuscript. All authors read and approved the final manuscript.

## Pre-publication history

The pre-publication history for this paper can be accessed here:

http://www.biomedcentral.com/1472-6920/14/172/prepub
